# Synthesis of high density aviation fuel with cyclopentanol derived from lignocellulose

**DOI:** 10.1038/srep09565

**Published:** 2015-03-31

**Authors:** Xueru Sheng, Ning Li, Guangyi Li, Wentao Wang, Jinfan Yang, Yu Cong, Aiqin Wang, Xiaodong Wang, Tao Zhang

**Affiliations:** 1 State Key Laboratory of Catalysis, Dalian Institute of Chemical Physics, Chinese Academy of Sciences, Dalian 116023, China; 2 Graduate University of Chinese Academy of Sciences, Beijing 10049, China; 3 Collaborative Innovation Center of Chemistry for Energy Materials (*i*ChEM)

## Abstract

For the first time, renewable high density aviation fuels were synthesized at high overall yield (95.6%) by the Guerbet reaction of cyclopentanol which can be derived from lignocellulose, followed by the hydrodeoxygenation (HDO). The solvent-free Guerbet reaction of cyclopentanol was carried out under the co-catalysis of solid bases and Raney metals. Among the investigated catalyst systems, the combinations of magnesium-aluminium hydrotalcite (MgAl-HT) and Raney Ni (or Raney Co) exhibited the best performances. Over them, high carbon yield (96.7%) of C_10_ and C_15_ oxygenates was achieved. The Guerbet reaction products were further hydrodeoxygenated to bi(cyclopentane) and tri(cyclopentane) over a series of Ni catalysts. These alkanes have high densities (0.86 g mL^−1^ and 0.91 g mL^−1^) and can be used as high density aviation fuels or additives to bio-jet fuel. Among the investigated HDO catalysts, the 35 wt.% Ni-SiO_2_-DP prepared by deposition-precipitation method exhibited the highest activity.

With the depletion of fossil energy and the increase of social concern about the environmental problems (such as CO_2_ and SO_2_ emissions) from the utilization of fossil energy, the catalytic conversion of renewable biomass to fuels^1^,^2^,^3^ and chemicals^4^,^5^,^6^,^7^,^8^ becomes a hot topic. Lignocellulose is the main component of agriculture wastes and forest residues. Jet fuel is one of the most often used transport fuels nowadays. Pioneered by the previous work of Dumesic^9^,^10^,^11^, Huber^12^,^13^, Corma^14^,^15^ and their groups, the synthesis of jet fuel range alkanes with the lignocellulose derived platform chemicals has drawn tremendous attention^16^,^17^,^18^,^19^,^20^.

So far, most of the reported lignocellulosic bio-jet fuels are primarily composed of linear or branched chain alkanes. These alkanes have good thermal stability and excellent combustion efficiency. However, their densities (~0.76 g mL^−1^) and volumetric heating values are lower than those of conventional jet fuels, because the latter is a mixture of chain alkanes and cyclic hydrocarbons. Due to the strong ring strain, cyclic hydrocarbons (Especially the polycyclic hydrocarbons) have higher densities and volumetric heating values than those of chain alkanes^21^. In real application, these chain alkanes must be blended with conventional jet fuel to meet the specification of aviation fuel. In order to solve this problem, it is imperative to develop new routes for the synthesis of jet fuel range cyclic hydrocarbons with lignocellulosic platform chemicals^22^,^23^,^24^,^25^,^26^.

Cyclopentanol is the aqueous-phase selective hydrogenation product of furfural which has been manufactured on an industrial scale by the hydrolysis-dehydration of the hemicellulose in agriculture wastes and forest residues^27^. In the recent work of Xiao's group^28^, it was found that cyclopentanol can be produced at high carbon yield (93.4%) by the aqueous-phase selective hydrogenation of furfural over Cu-MgAlO_3_ catalyst. Due to its cyclic structure, cyclopentanol can be used as a potential feedstock for the synthesis of high density polycyclic aviation fuel. However, there is no report about the synthesis of high density aviation fuel with cyclopentanol.

In this work, a mixture of bi(cyclopentane) and tri(cyclopentane) with high densities (0.86 g mL^−1^ and 0.91 g mL^−1^) was first prepared at high overall carbon yield (95.6%) by the Guerbet reaction of cyclopentanol, followed by the hydrodeoxygenation (HDO) over the SiO_2_ loaded Ni catalysts under solvent free conditions.

## Results and Discussion

### Guerbet reaction

**Figure 1:**
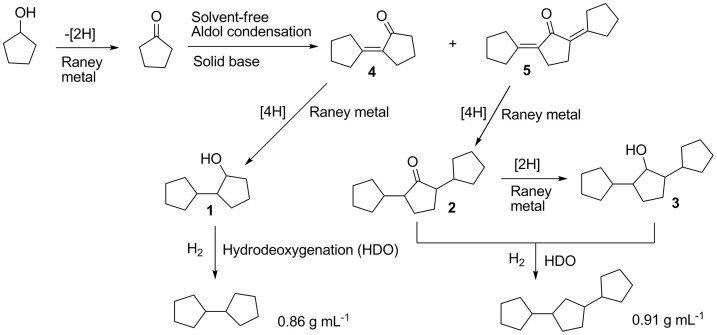
Reaction pathways for the synthesis of bi(cyclopentane) and tri(cyclopentane) with cyclopentanol as feedstock.

**Figure 2:**
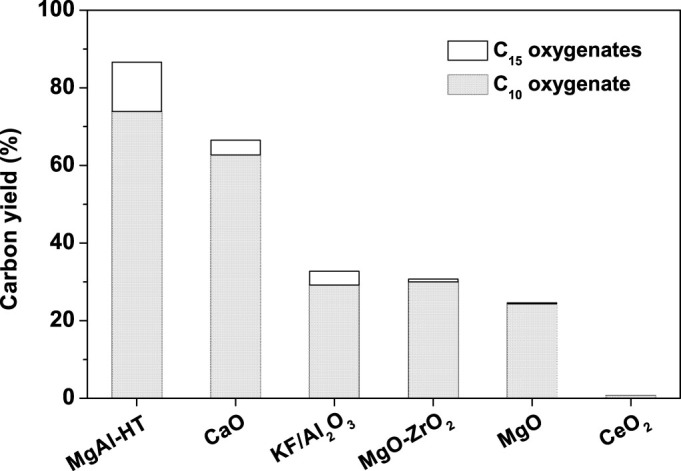
Effect of solid base on the carbon yields of C_10_ oxygenate (*i.e.* compound 1) and C_15_ oxygenates (*i.e.* compound 2 and 3). The reaction was conducted in a batch reactor at 443 K for 8 h with 4.0 g, 46.44 mmol cyclopentanol, 0.8 g solid base and 0.1 g Raney Ni.

The solvent-free Guerbet reaction of cyclopentanol was carried out under the co-catalysis of Raney metal and different solid base catalysts. From the analysis of GC-MS and NMR (see Supplementary Fig. S1–S4), 2-cyclopentyl-1-cyclopentanol (*i.e.* compound **1** in Fig. 1), 2,5-dicyclopentylcyclopentanone (*i.e.* compound **2** in Fig. 1) and 2,5-dicyclopentylcyclopentanol (*i.e.* compound **3** in Fig. 1) were identified as the main products. These oxygenates can be used as the precursors for the lignocellulosic high density jet fuel.

Figure 2 demonstrates the carbon yields of C_10_ (*i.e.* compound **1**) oxygenate and C_15_ oxygenates (*i.e.* compound **2** and **3**) obtained under the co-catalysis of Raney Ni and a series of solid bases. Among these catalysts, the combination of Raney Ni and magnesium-aluminium hydrotalcite (MgAl-HT) exhibited the best catalytic activity. Over it, 86.6% carbon yield of C_10_ and C_15_ oxygenates was achieved. The activity sequence for the investigated catalyst systems is: Raney Ni + MgAl-HT > Raney Ni + CaO > Raney Ni + KF/Al_2_O_3_ > Raney Ni + MgO-ZrO_2_ > Raney Ni + MgO > Raney Ni + CeO_2_. This sequence is consistent with the activity sequence of these solid bases for the self aldol condensation of cyclopentanone^22^.

**Figure 3:**
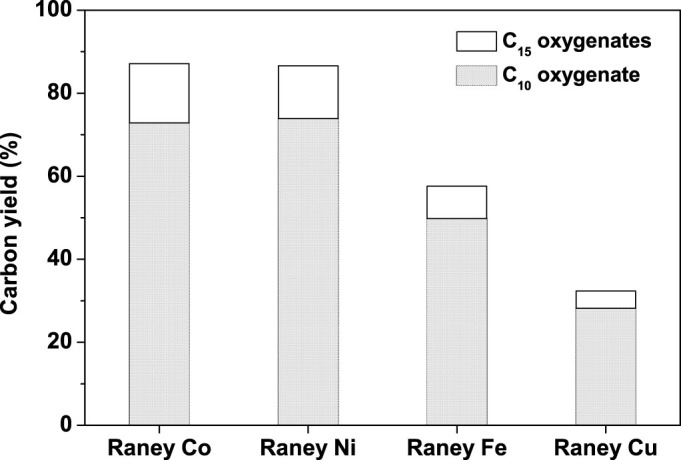
Effect of Raney metal on the carbon yields of C_10_ oxygenate (*i.e.* compound 1) and C_15_ oxygenates (*i.e.* compound 2 and 3). The reaction was conducted in a batch reactor at 443 K for 8 h with 4.0 g, 46.44 mmol cyclopentanol, 0.1 g Raney metals and 0.8 g MgAl-HT.

The influence of Raney metal was also studied. From the carbon yields of C_10_ oxygenate (*i.e.* compound **1**) and C_15_ oxygenates (*i.e.* compound **2** and **3**) demonstrated in Fig. 3, the combinations of Raney Ni (or Raney Co) with MgAl-HT exhibited the best performances among the investigated candidates. This result can be rationalized by the higher activity of Raney Ni (or Raney Co) for the dehydration of cyclopentanol.

**Figure 4:**
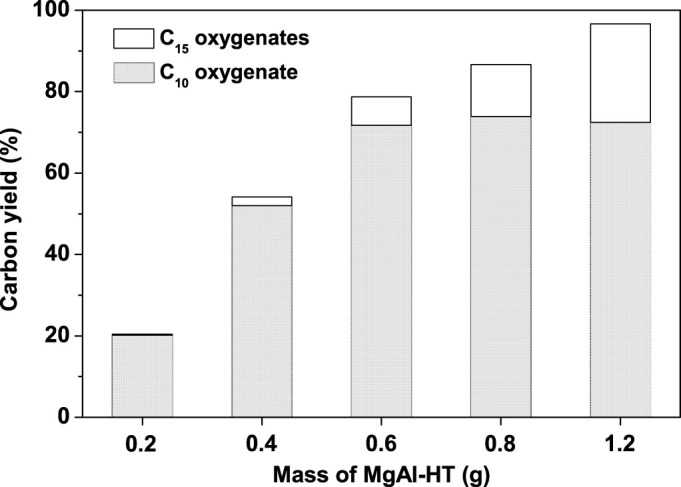
Effect of MgAl-HT dosage on the carbon yields of C_10_ oxygenate (*i.e.* compound 1) and C_15_ oxygenates (*i.e.* compound 2 and 3). The reaction was conducted in 443 K for 8 h with 4.0 g, 46.44 mmol cyclopentanol, 0.1 g Raney Ni and MgAl-HT.

Subsquently, the influence of MgAl-HT dosage on the carbon yields of C_10_ oxygenate (*i.e.* compound **1**) and C_15_ oxygenates (*i.e.* compound **2** and **3**) was also investigated. From the results shown in Fig. 4, the carbon yield of C_10_ oxygenate (*i.e.* compound **1**) increased with the increasing of MgAl-HT dosage from 0.2 g to 0.6 g, then leveled off with the further increment of MgAl-HT dosage. In contrast, the carbon yield of C_15_ oxygenates (*i.e.* compound **2** and **3**) (or the total carbon yield of C_10_ and C_15_ oxygenates) monotonously increased with the increment of MgAl-HT dosage from 0.2 g to 1.2 g. When 1.2 g MgAl-HT was used with 0.1 g Raney Ni catalyst, 72.5% carbon yield of C_10_ oxygenate (*i.e.* compound **1**) and 24.2% carbon yield of C_15_ oxygenates (*i.e.* compound **2** and **3**) (total carbon yield of C_10_ and C_15_ oxygenates: 96.7%) were achieved after reacting at 443 K for 8 h.

To get deeper insight of the individual roles of Raney metal and solid base in the Guerbet reaction of cyclopentanol, the catalytic performances of MgAl-HT and Raney Ni were investigated under the same reaction conditions. In the presence of MgAl-HT, no conversion of cyclopentanol was observed. Likewise, it was observed that Raney Ni only promoted the dehydrogenation of cyclopentanol (with cyclopentanone as the final product), neither C_10_ oxygenates nor C_15_ oxygenates was identified in the products. From these results, it can be seen that the synergy effect of solid base and Raney metal is necessary in the Guerbet reaction of cyclopentanol. The Raney metal promotes the dehydrogenation of cyclopentanol to cyclopentanone which further self-condenses to C_10_ and C_15_ oxygenates under the catalysis of solid base. As another function, the Raney metal also promotes the hydrogen transfer reactions between the cyclopentanol and the self aldol condensation products of cyclopentanone^7^,^29^, which may be the reason for the absence of α,β-unsaturated ketone (*i.e.* compound **4** and **5** in Fig. 1) in the Guerbet reaction products of cyclopentanol.

### Hydrodeoxygenation (HDO)

**Figure 5:**
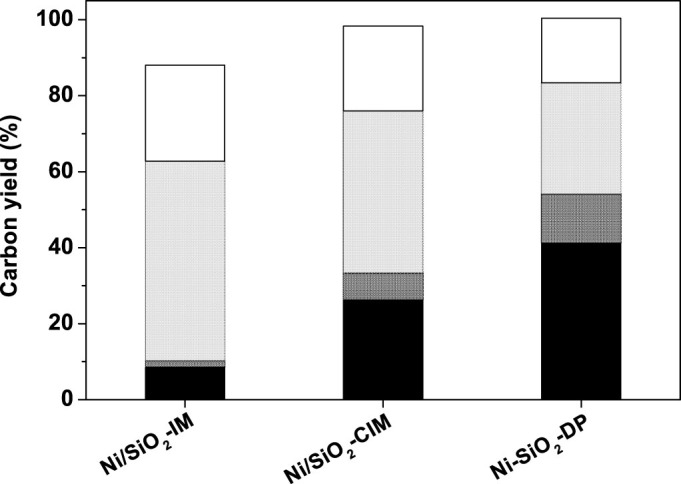
Carbon yields of bi(cyclopentane) (black bar), tri(cyclopentane) (dark grey bar), C_10_ oxygenates (light grey bar) and C_15_ oxygenates (white bar) over different Ni catalysts (the Ni contents in the catalysts are ~30 wt.%). Reaction conditions: 503 K, 6 MPa; 1.8 g of the Ni catalyst; Guerbet reaction products flow rate: 0.04 mL min^−1^; hydrogen flow rate: 120 mL min^−1^.

The solvent-free HDO of the Guerbet reaction products was carried over SiO_2_ loaded Ni catalysts. From the results shown in Fig. 5, it was found that the preparation method has strong influence on the HDO activity of Ni catalysts. Over the 30 wt.% Ni-SiO_2_-DP catalyst, evidently higher carbon yields to bi(cyclopentane) (41.4%) and tri(cyclopentane) (12.8%) were achieved by the HDO of Guerbet products of cyclopentanol at 503 K and 6 MPa. These alkanes have high densities (bi(cyclopentane): 0.86 g mL^−1^; tri(cyclopentane) 0.91 g mL^−1^)^30^. In real application, they can be used as high-density aviation fuels or additives to increase the volumetric heating value of bio-jet fuel or bio-diesel. The activity sequence of different Ni catalysts is: 30 wt.% Ni-SiO_2_-DP > 30 wt.% Ni/SiO_2_-CIM > 30 wt.% Ni/SiO_2_-IM. According to the results of H_2_-chemisorption (see Supplementary Table S2), there is evident correspondence between the activity of Ni catalysts and the metal dispersions over these catalysts. Therefore, the excellent performance of 30 wt.% Ni-SiO_2_-DP can be rationalized by the higher dispersion of Ni over this catalyst.

**Figure 6:**
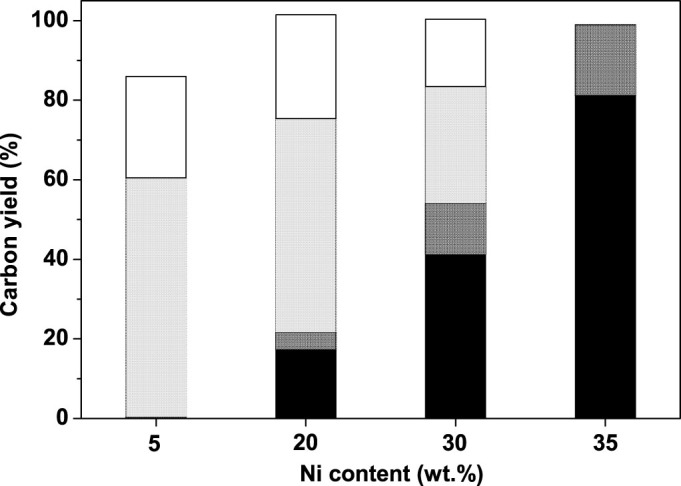
Carbon yields of bi(cyclopentane) (black bar), tri(cyclopentane) (dark grey bar), C_10_ oxygenates (light grey bar) and C_15_ oxygenates (white bar) over Ni-SiO_2_-DP as the function of Ni content. Reaction conditions: 503 K, 6 MPa; 1.8 g of the catalyst; Guerbet reaction products flow rate 0.04 mL min^−1^; hydrogen flow rate: 120 mL min^−1^.

The effect of Ni content on the catalytic performance of Ni-SiO_2_-DP was investigated. From the results shown in Fig. 6, the carbon yields of bi(cyclopentane) and tri(cyclopentane) increase with the increment of Ni content. The activity of Ni-SiO_2_-DP catalyst reaches the maximum value at the Ni content of 35 wt.%. Over the 35 wt.% Ni-SiO_2_-DP catalyst, the Guerbet products of cyclopentanol (*i.e.* compound **1**, **2** and **3** in Fig. 1) were completely converted, high carbon yield (98.9%) to bi(cyclopentane) and tri(cyclopentane) was achieved.

## Conclusions

Renewable high density aviation fuels were first synthesized at high overall yield (95.6%) by the solvent-free Guerbet reaction of cyclopentanol under the co-catalysis of Raney metal and solid base, followed by the hydrodeoxygenation (HDO) over Ni catalyst. Among the investigated catalyst systems, the combinations of Raney Ni (or Raney Co) and MgAl-HT exhibited the highest activity for the Guerbet reaction of cyclopentanol. The Guerbet products were further hydrodeoxygenated to bi(cyclopentane) and tri(cyclopentane) over a series of SiO_2_ loaded Ni catalysts. Among them, the 35 wt.% Ni-SiO_2_-DP catalyst prepared by deposition precipitation method exhibited the best catalytic performance.

## Methods

### Preparation of catalysts

Magnesium-aluminium hydrotalcite (MgAl-HT) with Mg/Al atomic ratio = 2 was prepared according to the literature^9^. Before being used in Guerbet reaction, the hydrotalcite was calcined in N_2_ flow at 723 K for 8 h. CaO, MgO and CeO_2_ were commercial available. MgO-ZrO_2_ was prepared according to the literature^31^. Before the activity test, the CaO, MgO, CeO_2_ and MgO-ZrO_2_ catalysts were calcined in N_2_ flow at 873 K for 3 h. KF/Al_2_O_3_ catalyst was prepared according to the literature^32^ by the incipient wetness impregnation of γ-Al_2_O_3_ with an aqueous solution of KF, followed by drying in N_2_ flow at 393 K overnight. The KF content in the KF/Al_2_O_3_ catalyst is 40% by weight (denoted as 40 wt.%). Raney Co, Raney Fe, Raney Cu and Raney Ni were commercial available. Before being used in the Guerbet reaction, these Raney metal catalysts were washed with deionized water for several times until the pH of water was 7.

The SiO_2_ loaded Ni catalysts used in the hydrodeoxygenation (HDO) of Guerbet reaction products were prepared by the methods of impregnation, complexion impregnation and deposition-precipitation, respectively. The detail information for the preparation of these Ni catalysts was described in supplementary information.

### Activity test

The solvent-free Guerbet reaction of cyclopentanol was conducted in a Teflon lined batch reactor. Typically, 4.0 g, 46.44 mmol cyclopentanol, 0.8 g solid base catalyst, 0.1 g Raney metal were used. Before reaction, the reactor was purged with argon for 3 times. After stirring at 443 K for 8 h, the reactor was quenched to room temperature. The products were taken out from the reactor, filtrated and analyzed by an Agilent 7890 A GC. The HDO of the Guerbet reaction products was carried out at 503 K in a 316 L stainless steel tubular flow reactor described in our previous work^18^,^22^,^33^,^34^,^35^. For each test, 1.8 g catalyst was used. Before the HDO process, the catalysts were reduced in-situ by hydrogen flow at 773 K for 2 h. After the reactor temperature was cooled down to 503 K and kept at this value for 0.5 h, the Guerbet reaction products (purified by vacuum distillation) were pumped into the reactor at 0.04 mL min^−1^ from the bottom along with hydrogen at a flow rate of 120 mL min^−1^. After coming out from the tubular reactor, the products became two phases in a gas-liquid separator. The gaseous products flowed through a back pressure regulator to maintain the system pressure at 6 MPa and were analyzed online by an Agilent 6890 N GC. The liquid products were drained periodically from the gas-liquid separator and analyzed by an Agilent 7890 A GC.

## Supplementary Material

Supplementary Information Supplementary Information
