# Supplementary material for: Synthesis of high density aviation fuel with cyclopentanol derived from lignocellulose
Source: Sci Rep. 2015 Mar 31;5:9565. doi: 10.1038/srep09565 (PMC5380329; doi:10.1038/srep09565)
Supplement: Supplementary Information [file srep09565-s1.pdf]

Supporting information

Synthesis of high density aviation fuel with cyclopentanol derived from lignocellulose

Xueru Sheng,[1,2] Ning Li,[1,3]* Guangyi Li,[1] Wentao Wang,[1] Jinfan Yang,[1,2] Yu Cong,[1] Aiqin Wang,[1,3] Xiaodong Wang,[1] and Tao Zhang [1,3]*

[1] *State Key Laboratory of Catalysis, Dalian Institute of Chemical Physics, Chinese Academy of Sciences, Dalian 116023, China.*

[2] *Graduate University of Chinese Academy of Sciences, Beijing 10049, China.*

[3] *Collaborative Innovation Center of Chemistry for Energy Materials (iChEM)*

*Corresponding author E-mail: taozhang@dicp.ac.cn (T. Zhang) or lining@dicp.ac.cn (N. Li)

Preparation of Ni catalysts:

The SiO_2 loaded Ni catalysts used in the hydrodeoxygenation (HDO) of Guerbet reaction products were prepared by the methods of impregnation, complexion impregnation and deposition-precipitation, respectively. To facilitate the comparison, the Ni contents in the impregnated catalysts were controlled as ~30 wt.% which is similar as the one in the 30 wt.% Ni-SiO_2-DP prepared by the deposition-precipitation (Supplementary Table S1 shows the specific Ni contents in different Ni catalysts which were measured by ICP analysis).

1) Impregnation (denoted as 30 wt.% Ni/SiO_2-IM): 8 g, 27.51 mmol $Ni(NO_3)_2$ $6H_2O$ was dissolved in 5 g H_2O. The solution was added into 3 g SiO_2 support, stirred and dried in 333 K until the excess water was evaporated. The resulting mixture was dried at 393 K overnight, calcined at 773 K for 4 h.

2) Complexion impregnation (denoted as 30 wt.% Ni/SiO_2-CIM): 8 g, 27.51 mmol $Ni(NO_3)_2$ $6H_2O$ and 1.71 g, 27.55 mmol ethylene glycol was added into 3.3 g H_2O, the solution was added into 3 g SiO_2 support, stirred and dried in 333 K until the excess water was evaporated, The resulting mixture was dried at 393 K overnight, calcined at 773 K for 4 h.

3) Deposition precipitation (denoted as 30 wt.% Ni-SiO_2-DP): The 30 wt.% Ni-SiO_2-DP were prepared by the according to the literature[1]. The 500 mL aqueous solution prepared by dissolving 15.26 g, 52.47 mmol $Ni(NO_3)_2 \cdot 6H_2O$ in deionized water was divided into two parts. 9.45 g, 157.34 mmol urea was added to one part of this solution (100 mL). Then, the $Ni(NO_3)_2 \cdot 6H_2O$ solution with urea was added drop

wise to the rest $Ni(NO_3)_2·6H_2O$ solution (400 mL) together with 3.8 g SiO_2 support and 0.48 mL 65 wt.% HNO_3 under vigorous stirring at 353 K. After the precipitation process, the suspension was rapidly heated to 363 K and stirred at this temperature for 10 h. The solid was filtered, washed to neutral, dried overnight and calcined in air at 773 K for 4 h. For comparison, the Ni-SiO_2-DP catalysts with other Ni contents were prepared analogously by changing the amount of $Ni(NO_3)_2·6H_2O$, urea and 65 wt.% HNO_3 solution.

Characterization of catalysts:

1. XRD

XRD patterns of different Ni catalysts were recorded with a PANalytical X'Pert-Pro powder X-ray diffractometer, using Cu Kα monochromatized radiation (λ = 0.1541 nm) at a scan speed of 5 °min^{-1}. Before the tests, the catalysts were reduced by hydrogen flow at 773 K for 2 h. From the results shown in supplementary Fig. S5, only the peaks of metallic Ni and SiO_2 support were observed in the XRD patterns of different Ni catalysts. No peak for nickel oxide was observed.

2. H_2 chemisorption

The average sizes, metal dispersions and metallic surface areas of Ni catalysts were characterized by H_2 chemisorption which was carried out with a Micromeritics Autochem II 2920 automated chemisorption analyzer. Before each test, the sample was reduced in 10% H_2/Ar flow at 773 K for 2 h. Subsequently, the sample was purged with Ar flow at 783 K for 0.5 h and cooled down in Ar flow to 323 K. After the stabilization of baseline, the H_2 adsorption was carried out by the pulse adsorption

of 10% H_2/Ar at 323 K. From the results listed in supplementary Table S2, the metal dispersions and metallic surface areas of Ni over different catalysts decrease in the order of 30 wt.% Ni-SiO_2-DP > 30 wt.% Ni/SiO_2-CIM > 30 wt.% Ni/SiO_2-IM. According to these results, two conclusions can be drawn: 1) the deposition precipitation method is better than impregnation method to get a well dispersed Ni catalyst; 2) the presence of ethylene glycol is beneficial to the dispersion of Ni over SiO_2 support, which can be rationalized by the complex effect [2].

3. TEM and HRTEM

The TEM images of the 30 wt.% Ni/SiO_2-IM and the 30 wt.% Ni/SiO_2-CIM catalysts were obtained with a transmission electron microscopy (TEM, FEI Tecnai G2 Spirit) operated at an accelerating voltage of 120 kV. The HRTEM images of 30 wt.% Ni-SiO_2-DP catalyst was obtained with a high revolution transmission electron microscopy (HRTEM, JEM-2100F) operated at an accelerating voltage of 200 kV. Before the tests, the Ni catalysts were reduced by hydrogen flow at 773 K for 2 h. From supplementary Fig. S6, it is clear that the Ni particles on 30 wt.% Ni-SiO_2-DP are evidently smaller than those over 30 wt.% Ni/SiO_2-IM and 30 wt.% Ni/SiO_2-CIM. This result is consistent with what we have observed by H_2 chemisorption.

Method for the calculation of carbon yield in hydrodeoxygenation (HDO) step:

Carbon yield of bi(cyclopentane) (%) = Sum of carbon in the bi(cyclopentane) detected from the liquid phase HDO product/Carbon fed into the reactor $\times$100%

Carbon yield of tri(cyclopentane) (%) = Sum of carbon in the tri(cyclopentane) detected from the liquid phase HDO product/Carbon fed into the reactor $\times$100%

Carbon yield of C_{10} oxygenates (%) = Sum of carbon in the C_{10} oxygenates detected from the liquid phase HDO product/Carbon fed into the reactor $\times$100%

Carbon yield of C_{15} oxygenates (%) = Sum of carbon in the C_{15} oxygenates detected from the liquid phase HDO product/Carbon fed into the reactor $\times$100%

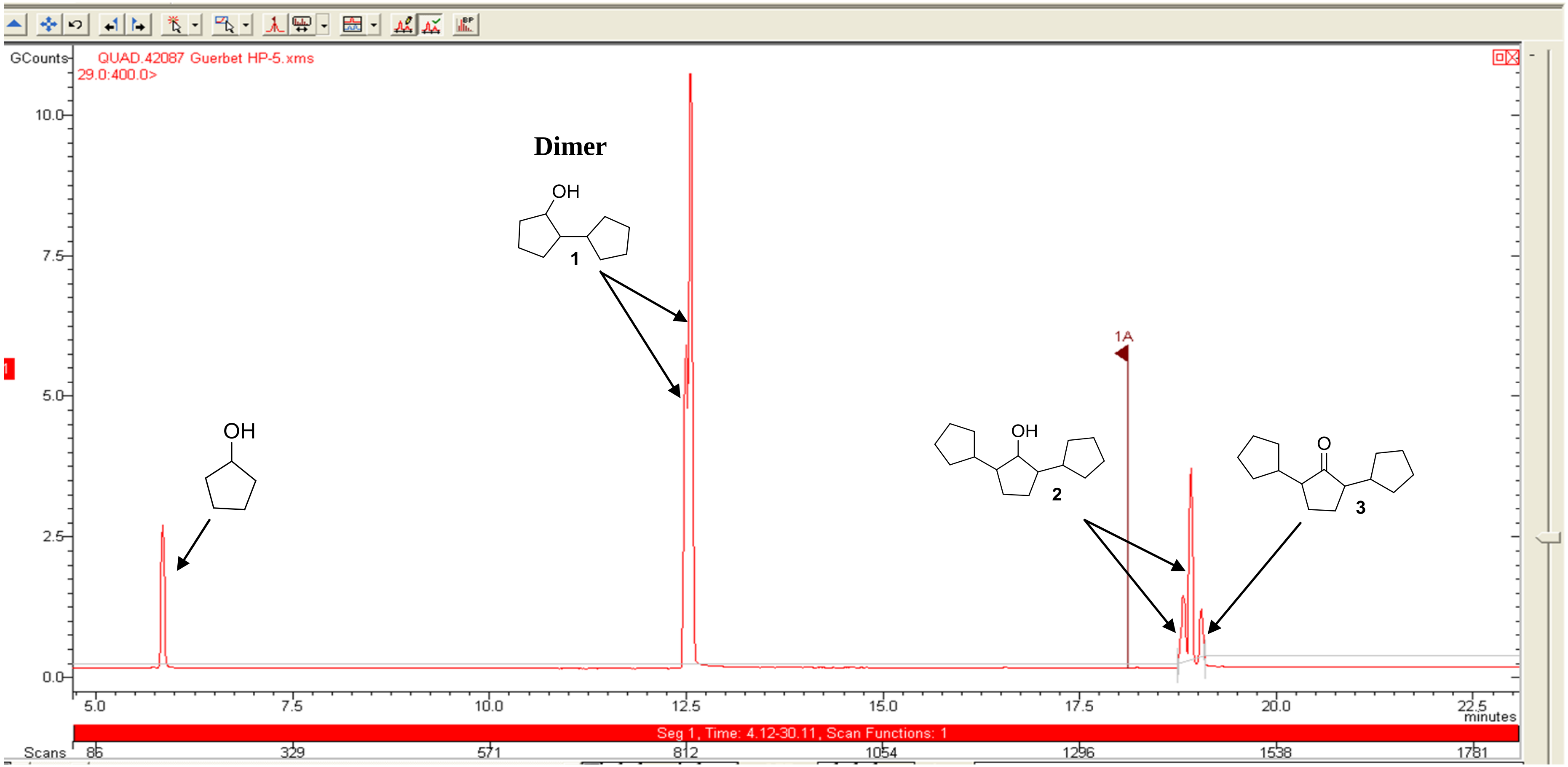

Figure S1 | GC-MS spectrum of the liquid products from the Guerbet reaction of cyclopentanol. Reaction conditions: 443 K, 8 h, 4.0 g 46.44 mmol cyclopentanol, 1.2 g MgAl-HT and 0.1 g Raney Ni.

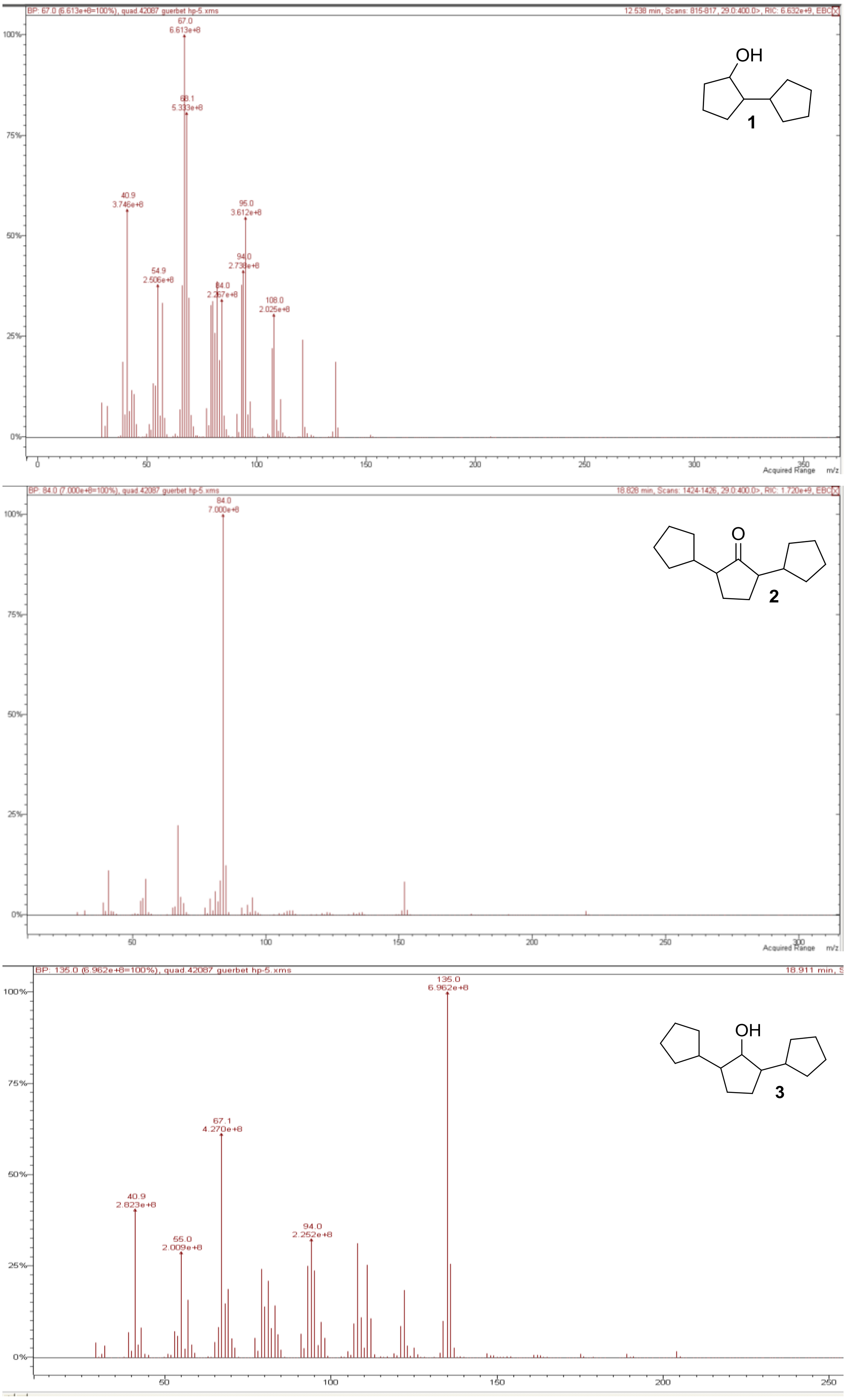

Figure S2 | Mass spectrograms of compound 3, 4 and 5 from the Guerbet reaction of cyclopentanol. Reaction conditions: 443 K, 8 h, 4.0 g, 46.44 mmol cyclopentanol, 1.2 g MgAl-HT and 0.1 g Raney Ni.

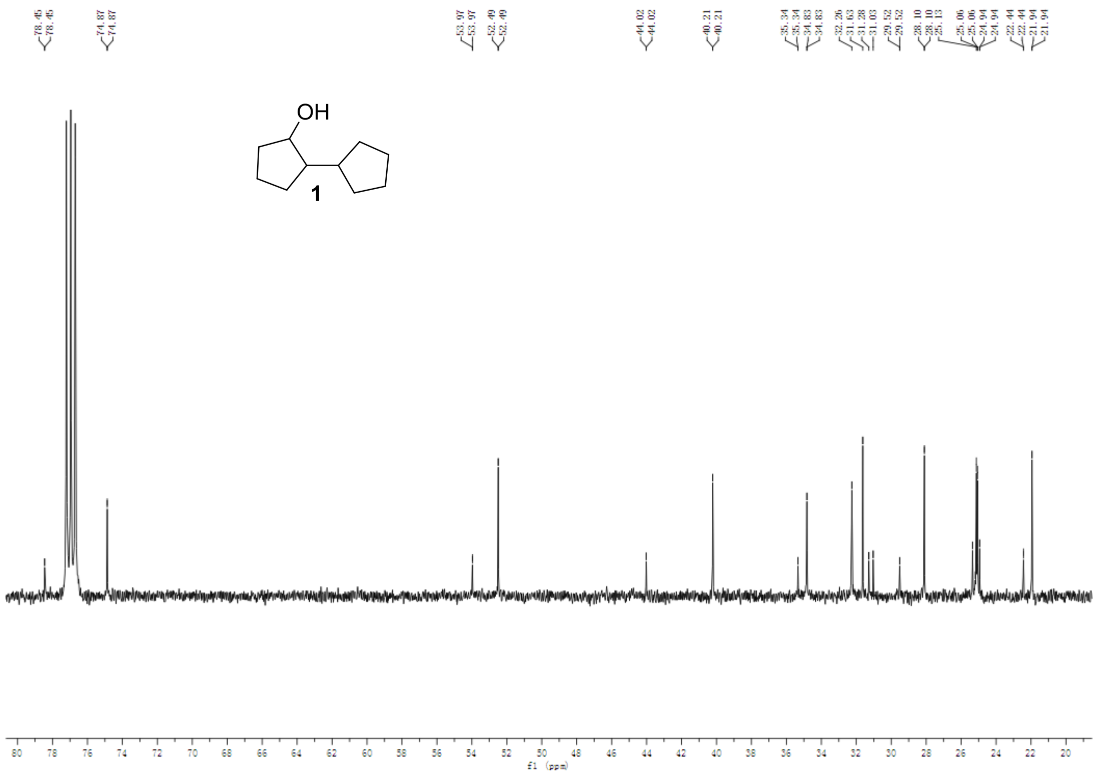

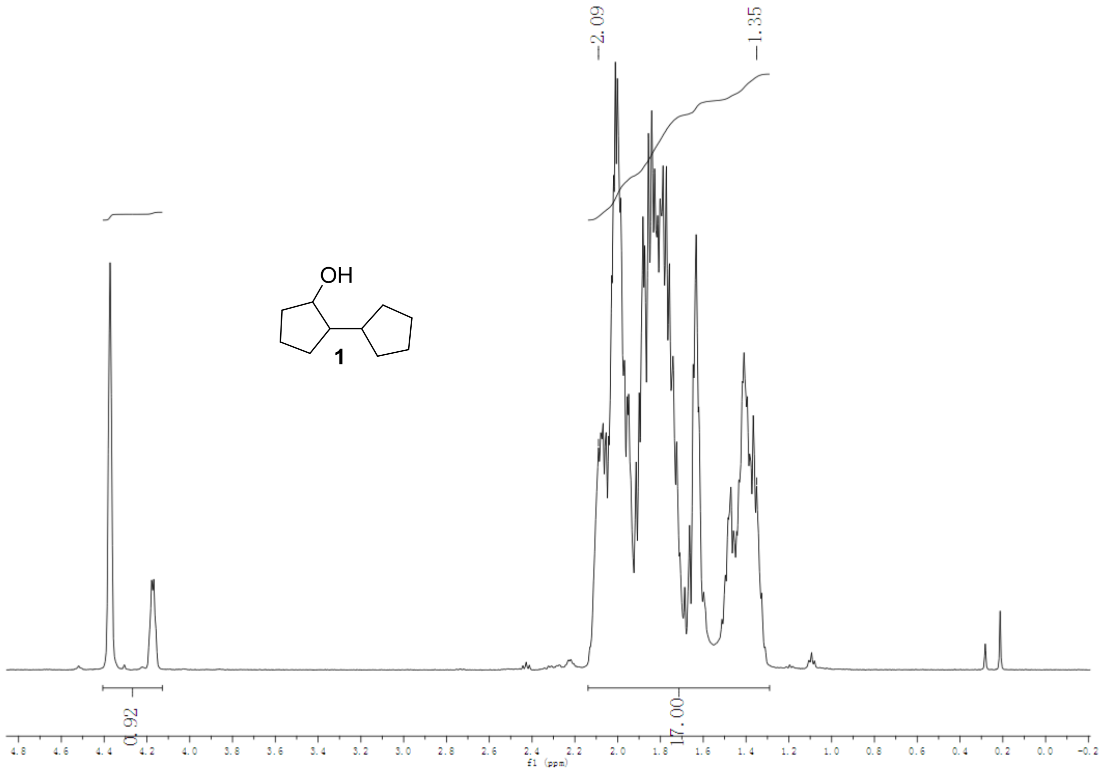

Figure S3 | ^{13}C and ^{1}H NMR spectra of the compound 1 which was produced by the Guerbet reaction of cyclopentanol.

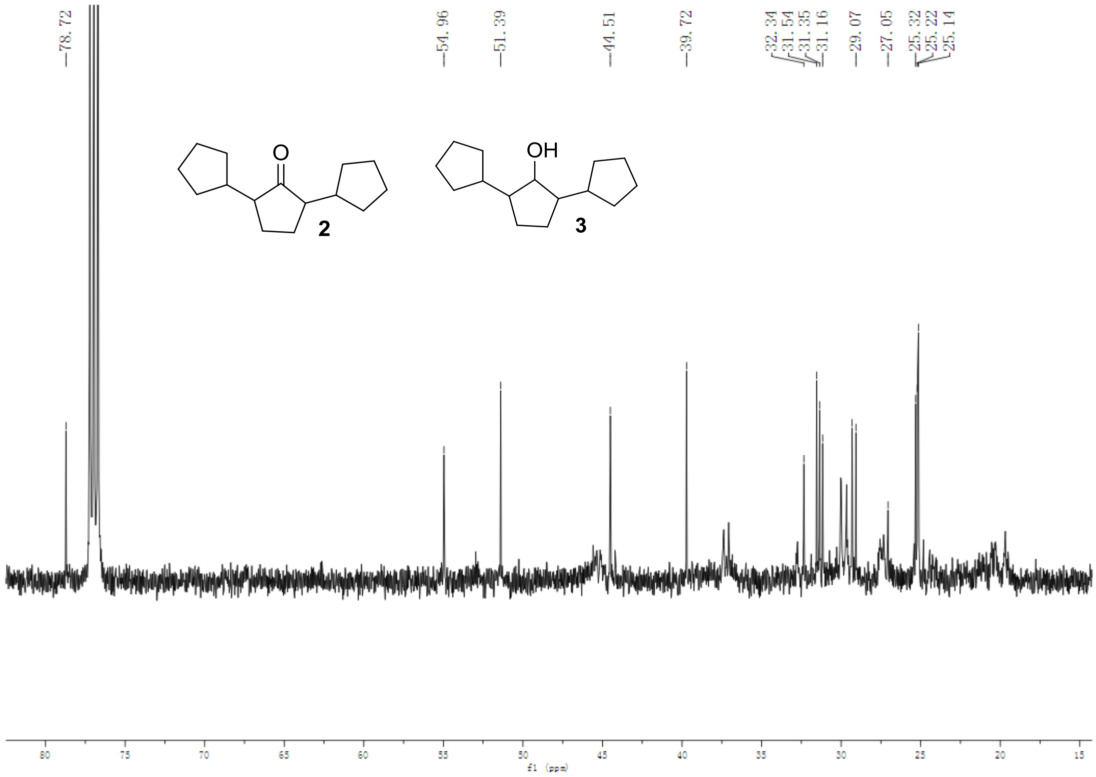

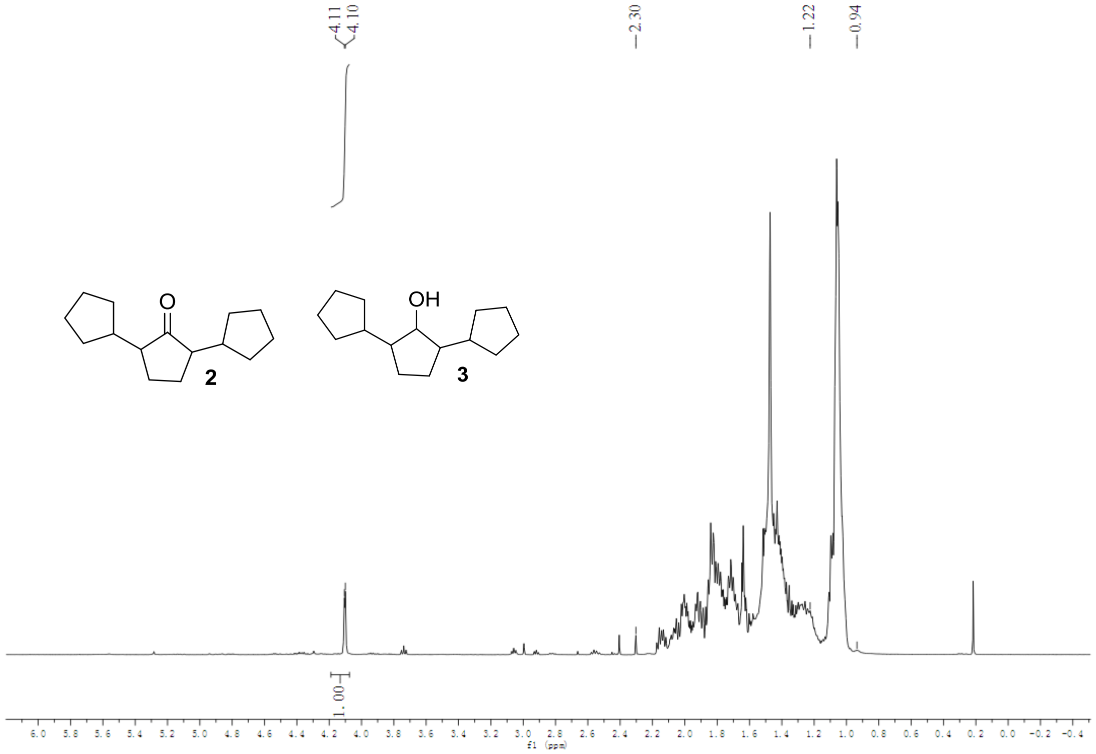

Figure S4 | ^{13}C and ^{1}H NMR spectra of the compound 2 and 3 which were produced by the Guerbet reaction of cyclopentanol.

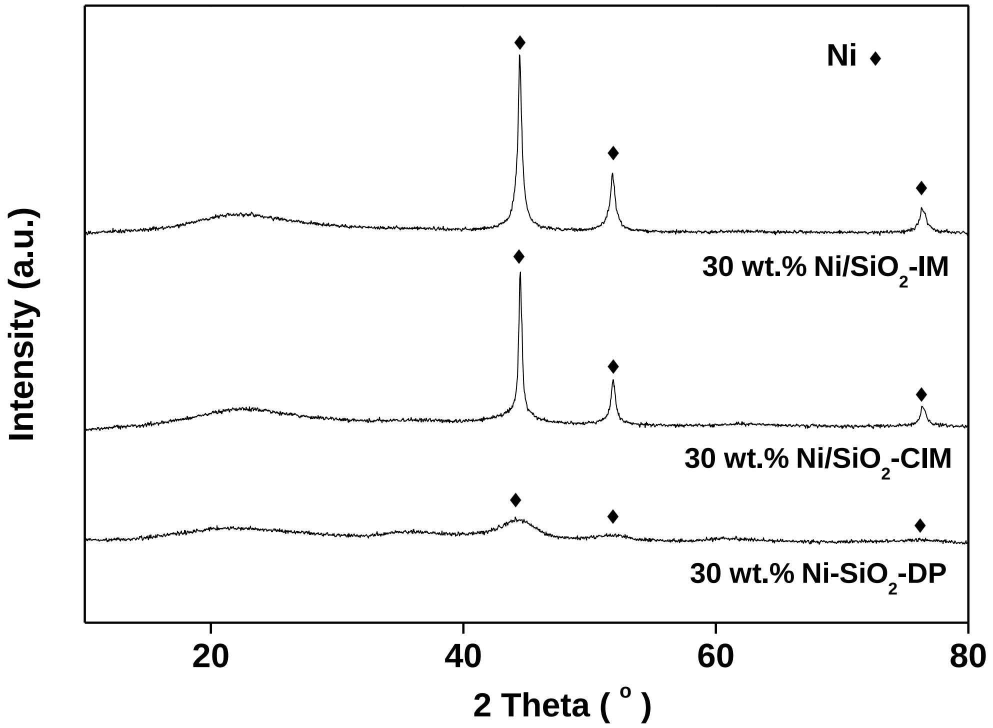

Figure S5 | XRD patterns of SiO_2 loaded Ni catalysts prepared by different methods.

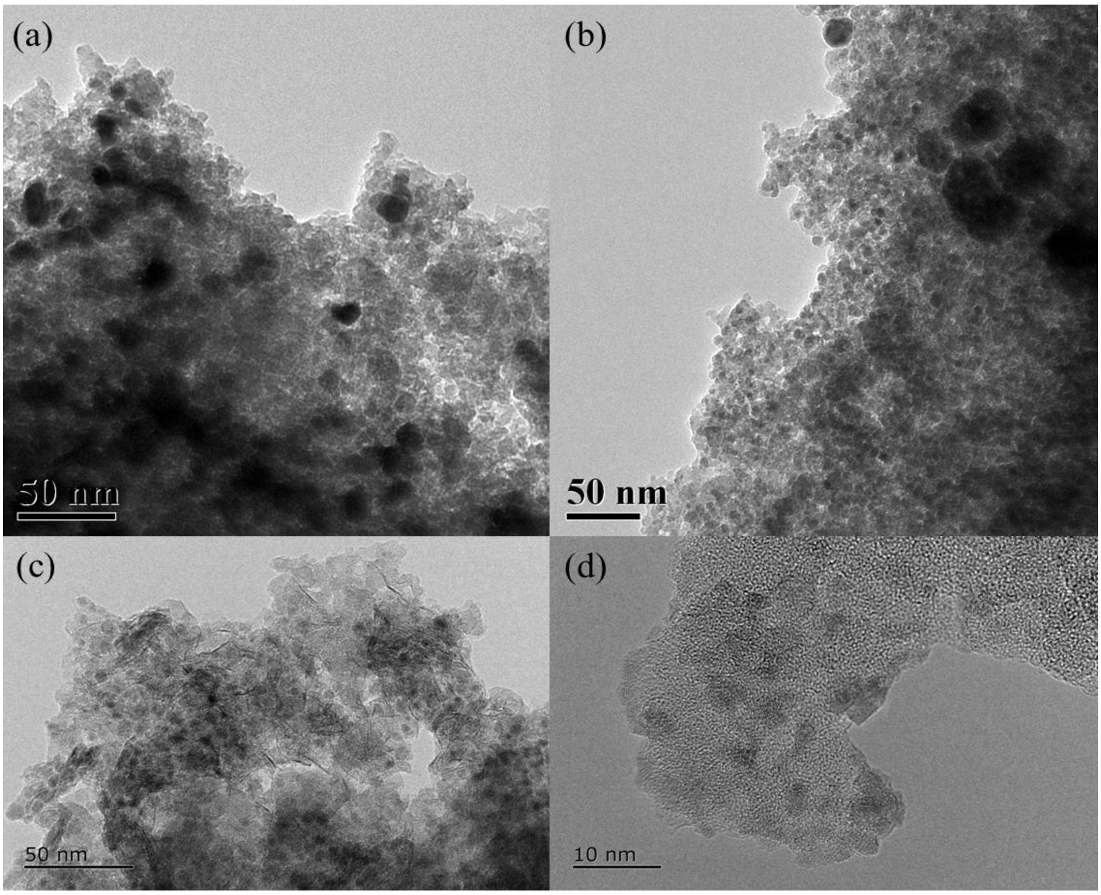

Figure S6 | TEM images of (a): 30 wt. % Ni/SiO_2-IM; (b): 30 wt. % Ni/SiO_2-CIM and HRTEM images of (c), (d): 30 wt. % Ni-SiO_2-DP.

Table S1 | The actual Ni contents in the HDO catalysts.

Catalyst	Ni content (wt.%) [a]
35 wt.% Ni-SiO_2-DP	35.9
30 wt.% Ni-SiO_2-DP	31.2
20 wt.% Ni-SiO_2-DP	19.4
5 wt.% Ni-SiO_2-DP	5.3
30 wt.% Ni/SiO_2-IM	29.3
30 wt.% Ni/SiO_2-CIM	31.1

a: The actual Ni contents in catalysts were measured by Thermo IRIS Intrepid II inductively coupled plasma (Perkin-Elmer Optima 7300DV).

Table S2 | Average sizes of Ni particles, metal dispersions and metallic surface areas on different Ni catalysts [a].

Catalyst	Average sizes (nm)	Metal dispersion (%)	Metallic surface area (m^2 g^{-1})
30 wt.% Ni/SiO_2-IM	25.1	3.36	6.06
30 wt.% Ni/SiO_2-CIM	13.0	6.49	12.39
30 wt.% Ni-SiO_2-DP	9.0	9.38	19.49

a： Calculated from the results of H_2 chemisorption.

References

1 He, J. Y., Zhao, C. & Lercher, J. A. Ni-Catalyzed Cleavage of Aryl Ethers in the Aqueous Phase. *J. Am. Chem. Soc.* **134**, 20768-20775 (2012).

2 Qiu, S. *et al.* A simple method to prepare highly active and dispersed Ni/MCM-41 catalysts by co-impregnation. *Catal. Commun.* **42**, 73-78, (2013).
